# Longitudinal Changes in Somatosensory Function and Their Association with Pain Trajectory Groups in a Portuguese Cancer Center Cohort

**DOI:** 10.3390/biomedicines14081840

**Published:** 2026-08-15

**Authors:** Ana Sofia Martins, Mariana Ferreira, Cláudia Vieira, Otília Romano, Rosário Rodrigues, Diana Ramada, Catarina Castro, Ana Paula Moreira, María Teresa Carrillo-de-la-Peña, Ana Luísa Teixeira, Rui Medeiros

**Affiliations:** 1 Molecular Oncology and Viral Pathology Group, IPO Porto Research Center (CI-IPO Porto), Portuguese Oncology Institute of Porto (IPO Porto), 4200-072 Porto, Portugal; i13730@ipoporto.min-saude.pt (A.S.M.); mariana.f.ferreira@ipoporto.min-saude.pt (M.F.); catarina2castro@hotmail.com (C.C.); 2 RISE—Health Research Network, 4200-319 Porto, Portugal; diana.ramada@ipoporto.min-saude.pt; 3 Porto Comprehensive Cancer Centre Raquel Seruca (Porto.CCC), 4200-072 Porto, Portugal; 4 Research Department, Portuguese League Against Cancer, Northern Branch (LPCC-NRN), 4200-172 Porto, Portugal; 5 Department of Oncology, Portuguese Oncology Institute of Porto (IPO Porto), 4200-072 Porto, Portugal; claudia.vieira@ipoporto.min-saude.pt; 6 Physical and Rehabilitation Medicine Clinic, Portuguese Oncology Institute of Porto (IPO Porto), 4200-072 Porto, Portugal; otilia.romano@ipoporto.min-saude.pt; 7 Department of Medicine, Portuguese Oncology Institute of Porto (IPO Porto), 4200-072 Porto, Portugal; mariarodrigues@ipoporto.min-saude.pt; 8 Clinical Research Unit, IPO Porto Research Center (CI-IPO Porto), Portuguese Oncology Institute of Porto (IPO Porto), 4200-072 Porto, Portugal; 9 Oncology Nursing Research Unit, IPO Porto Research Center (CI-IPO Porto), Portuguese Oncology Institute of Porto (IPO Porto), 4200-072 Porto, Portugal; 10 Nursing Management Department, Portuguese Oncology Institute of Porto (IPO Porto), 4200-072 Porto, Portugal; paula.moreira@ipoporto.min-saude.pt; 11 Brain and Pain (BaP) Lab, Departamento de Psicoloxía Clínica y Psicobioloxía, Facultade de Psicoloxia, Universidade de Santiago de Compostela, Campus Vida, 15782 Santiago de Compostela, A Coruña, Spain; mteresa.carrillo@usc.es; 12 School of Medicine and Biomedical Sciences (ICBAS), University of Porto, 4050-313 Porto, Portugal; 13 Biomedical Research Center (CEBIMED), Faculty of Health Sciences, Fernando Pessoa University (UFP), 4249-004 Porto, Portugal; 14 Faculty of Medicine, University of Porto (FMUP), 4200-319 Porto, Portugal

**Keywords:** cancer pain, quantitative sensory testing, somatosensory disorders, pain measurement, sensory thresholds, longitudinal studies

## Abstract

**Background:** Cancer-related pain involves complex peripheral and central mechanisms. Quantitative sensory testing (QST) assesses somatosensory function and provides insights into pain mechanisms. However, longitudinal evidence in oncological populations remains limited. This study investigated longitudinal changes in somatosensory function and their relationship with pain intensity and pain trajectory groups in a Portuguese cancer cohort. **Methods:** This longitudinal study was conducted within the international PAINLESS project and included participants from the Portuguese clinical unit. A total of 56 cancer patients were assessed at diagnosis (baseline, T0), before treatment, and at 6-month follow-up (T6). Pain intensity was measured using the Numeric Rating Scale (NRS), and somatosensory function was assessed using standardized thermal QST, including heat pain threshold (HPT), cold detection threshold (CDT), cold pain threshold (CPT), temporal summation of pain (TSP), and conditioned pain modulation (CPM). Non-parametric analyses evaluated longitudinal changes, correlations between pain and QST measures, differences according to clinically relevant pain (NRS < 4 vs. ≥ 4), and differences across clinically meaningful pain trajectory groups. **Results:** Thermal sensory thresholds changed significantly, with decreased HPT (*p* = 0.025) and increased CDT (*p* < 0.001) and CPT (*p* = 0.005). At T0, pain intensity was negatively correlated with CDT (*r_s_* = − 0.371, *p* = 0.009), whereas at T6 it was positively correlated with CPT (*r_s_* = 0.401, *p* = 0.006). Longitudinal changes in pain intensity were also associated with changes in CPT (*r_s_* = 0.337, *p* = 0.029). Trajectory analysis demonstrated significant baseline CDT differences across pain trajectory groups (*p* = 0.028), with higher CDT values in the pain increase group than in the persistent pain group (adjusted *p* = 0.035). No significant longitudinal changes were observed in central pain modulatory measures (TSP and CPM). **Conclusions:** Altered thermal sensory processing, particularly cold-related measures, was associated with pain intensity and trajectories during the first six months after diagnosis. While central pain modulation remained stable, CDT and CPT showed consistent relationships with clinically relevant pain outcomes. These findings support the potential utility of thermal QST for characterizing sensory profiles and pain trajectory groups in cancer-related pain; being important, further longitudinal studies are needed to determine the predictive value of these measures.

## 1. Introduction

Cancer-related pain is a frequent and complex clinical problem, with a substantial impact on patients’ quality of life, functional status, and psychological well-being throughout the course of the disease and treatment trajectory [1]. Recent evidence indicates that approximately 44.5% of cancer patients report pain and that about 30.6% experience moderate to severe pain, reflecting a reduction from the 38.0% prevalence reported in the previous decade [2]. However, this burden is significantly higher in subgroups with advanced disease, where the prevalence of self-reported pain can reach 91.6%, despite its limited documentation in clinical records (15.4%) [3].

Clinical assessment of pain in oncology relies predominantly on self-reported measures, such as the Numeric Rating Scale (NRS, 0–10) [4]. However, the clinical interpretation of NRS values depends on their relationship with perceived severity and functional interference [5]. Recent evidence demonstrates considerable variability in the cut-off values used to classify pain intensity across studies [6]. Nevertheless, a threshold of NRS ≥ 4 is commonly considered to represent at least moderate pain [6] and has been associated with greater pain-related interference and functional impact, and pain levels exceeding what is generally considered tolerable [7].

In this context, there has been increasing interest in approaches that allow for the characterization of pain-related sensory processing beyond self-reported intensity, in order to clarify the underlying somatosensory alterations. Quantitative Sensory Testing (QST) is a psychophysical methodology widely used to assess somatosensory system function, enabling the characterization of sensory thresholds and dynamic pain modulation processes through standardized stimuli [8]. Over recent decades, QST has been extensively applied across different chronic pain conditions, demonstrating utility in identifying distinct sensory profiles and alterations in facilitatory and inhibitory nociceptive processes [9]. Therefore, it may help clinicians recognize patients at increased risk of developing persistent or treatment-related pain, thereby supporting earlier interventions and more individualized pain management strategies [10,11].

Thermal QST allows for the functional assessment of somatosensory pathways involved in the detection and processing of both innocuous and nociceptive thermal stimuli [12]. Cold detection threshold (CDT) is primarily mediated by thinly myelinated Aδ fibers and cold-sensitive transient receptor potential (TRP) channels, particularly TRPM8. In contrast, cold pain threshold (CPT) and heat pain threshold (HPT) additionally involve nociceptive C fibers and thermosensitive receptors such as TRPA1 and TRPV1 [13,14,15]. Therefore, alterations in thermal thresholds may reflect changes in peripheral sensory function, small-fiber integrity, or altered nociceptive processing mechanisms. As CDT, CPT, and HPT assess distinct components of thermal sensation and nociception, they provide a broader functional assessment of peripheral somatosensory pathways.

Persistent nociceptive input and neuroimmune interactions may contribute to central nervous system plasticity, resulting in changes in sensory processing over time [16,17]. Dynamic QST measures, including temporal summation of pain (TSP) and conditioned pain modulation (CPM), provide indirect information on pain modulation mechanisms and therefore complement thermal QST by assessing central pain processing. TSP refers to the progressive increase in perceived pain during repeated application of identical noxious stimuli and is commonly interpreted as a psychophysical correlate of central sensitization and spinal “wind-up” phenomena [17]. In contrast, CPM assesses the function of endogenous descending inhibitory pathways by quantifying changes in the response to a test stimulus in the presence of a heterotopic conditioning stimulus [18]. Previous studies have shown that alterations in TSP and CPM are associated with persistent pain states and greater clinical severity across different populations, supporting their relevance as surrogate markers of altered pain modulation [19,20]. Nevertheless, as psychophysical proxy measures, TSP and CPM should be interpreted in light of their known methodological limitations, including the influence of cognitive and psychological factors on pain perception and the inconsistent test–retest reliability reported for CPM [21,22]. Together, thermal and dynamic QST measures provide complementary information on peripheral somatosensory function and central pain modulation, enabling a more comprehensive characterization of somatosensory alterations associated with cancer-related pain.

Despite the growing use of QST in chronic pain research, its application in the oncological context remains relatively heterogeneous [23,24]. Most studies employing QST in oncological settings, particularly in chemotherapy-induced peripheral neuropathy, are based on cross-sectional designs in which sensory parameters are assessed at a single time point, frequently after pain onset or therapeutic exposure [24,25]. Although these approaches provide important insights into pain-related sensory alterations, they limit the understanding of temporal changes in nociceptive modulation mechanisms. Furthermore, cancer-related pain is increasingly recognized as a multidimensional process involving both peripheral and central mechanisms [16,17,26]. However, longitudinal evidence characterizing dynamic changes in somatosensory processing throughout the disease and treatment trajectory remains limited [23].

Although pain intensity and somatosensory function represent distinct dimensions of the pain experience, examining their relationship may provide insight into the sensory mechanisms associated with clinically relevant pain and their evolution over time. Longitudinal assessment of QST parameters from cancer diagnosis to a 6-month follow-up may therefore contribute to a more comprehensive understanding of somatosensory alterations during cancer treatment and help identify sensory profiles associated with different patterns of pain evolution. Therefore, the aim of the present study is to characterize longitudinal changes in somatosensory function and to investigate the association between QST parameters, pain intensity, and pain trajectory groups over time in a Portuguese cancer center cohort.

## 2. Materials and Methods

### 2.1. Study Design and Participants

This longitudinal observational study was conducted at the Portuguese Institute of Oncology of Porto (IPO Porto) within the framework of the international PAINLESS project (Grant Agreement No. 101057367). Data collection began on 16 November 2023, and all participants provided written informed consent before participation.

Eligible participants were adults (≥18 years old) with a recent diagnosis (<6 months) of primary breast, lung, pancreatic, or colorectal cancer with metastases or not, who had not yet initiated cancer treatment, were able to self-report pain, and had an estimated life expectancy of at least 12 months. Exclusion criteria included pregnancy or lack of effective contraception in women of fertile age, neurological or psychiatric diseases (except anxiety and depression), unstable medical conditions, history of neurosurgery, traumatic brain injury with loss of consciousness; and/or cortical lesions, and a history of non-malignant chronic pain.

Patients were evaluated at two time points: baseline (T0), corresponding to cancer diagnosis prior to any therapeutic intervention, and 6-month follow-up (T6). At both assessments, patients rated their cancer-related pain intensity using an 11-point Numeric Rating Scale (0 = no pain, 10 = worst imaginable pain) and somatosensory function was evaluated through a standardized quantitative sensory testing protocol.

A total of 56 patients were included in the study. Although no formal a priori sample size calculation was performed because of the observational design and consecutive recruitment strategy, an exploratory sample size calculation indicated that approximately 58 participants would be required to detect a moderate correlation (*r* = 0.36) with 80% power at a two-sided significance level of 0.05, which was comparable to the final sample of 56 participants. Information on demographic and clinical variables was collected from the study database, including age, gender, type of cancer, cancer stage, and oncological treatments received during follow-up, namely chemotherapy, hormone therapy, immunotherapy, targeted therapy, radiotherapy, and oncological surgery.

The study population is predominantly composed of women (91.1%) with the majority of participants diagnosed with breast cancer (80.4%), reflecting the characteristics of the patients consecutively recruited from the participating Portuguese cancer center.

### 2.2. Quantitative Sensory Testing Protocol

Somatosensory function was assessed using a standardized thermal QST protocol applied to the volar forearm, as previously described [10]. The protocol included the assessment of heat pain threshold (HPT), cold detection threshold (CDT), cold pain threshold (CPT), temporal summation of pain (TSP), and conditioned pain modulation (CPM).

For HPT, CDT, and CPT, three repeated trials were obtained and averaged for analysis. Thermal thresholds were expressed as absolute temperature values (°C). TSP and CPM were assessed using an individualized thermal stimulus calibrated to a pain intensity of 5/10 on the NRS (Pain5). For TSP assessment, participants rated the pain intensity elicited by a single heat stimulus (Stim1) and by the last stimulus of a sequence of 10 consecutive heat stimuli (Stim10). The TSP effect was calculated as: TSP effect=Stim10−Stim1. For CPM assessment, participants rated the intensity of a heat test stimulus before (${\mathrm{Stim}}_{\mathrm{pre}}$) and after (${\mathrm{Stim}}_{\mathrm{post}}$) exposure to a conditioning stimulus consisting of hand contact with a cold plate. The CPM effect was calculated as: $\mathrm{CPM}\;\mathrm{effect}={\mathrm{Stim}}_{\mathrm{post}}-{\mathrm{Stim}}_{\mathrm{pre}}$.

### 2.3. Pain-Related Variables

Although cut-points for cancer pain severity are not entirely uniform across studies [6], pain severity can be categorized as no pain (0), mild pain (1–3), moderate pain (4–6), and severe pain (7–10) [27]. Previous evidence indicates that a cut-point of 4 is the most frequently recommended boundary between mild and moderate cancer pain and is associated with greater pain-related interference and need for clinical management [6,7].

Accordingly, in addition to analyzing NRS scores as a continuous variable, pain intensity was dichotomized as NRS < 4 and NRS ≥ 4 to distinguish no/mild pain from clinically relevant pain (i.e., at least moderate pain). This approach was considered more appropriate for subgroup analyses because a more granular categorization would have generated small subgroup sizes, reducing statistical power and interpretability.

To explore pain trajectory groups across time, a four-group pain trajectory variable was created based on the combination of pain status at T0 and T6. Participants were classified as follows: (i) stable low pain, representing participants who remained below the threshold for clinically relevant pain at both assessments (NRS < 4 at T0 and T6); (ii) pain increase, representing participants whose pain increased from non-clinically relevant to clinically relevant levels (NRS < 4 at T0 and NRS ≥ 4 at T6); (iii) pain decrease, representing participants whose pain decreased from clinically relevant to non-clinically relevant levels (NRS ≥ 4 at T0 and NRS < 4 at T6); and (iv) persistent pain, representing participants who reported clinically relevant pain at both assessments (NRS ≥ 4 at T0 and T6). This categorization was used to explore whether somatosensory profiles assessed by QST differed according to clinically meaningful pain trajectory groups over time.

### 2.4. Derived Longitudinal Variables

For longitudinal analyses, change scores (Δ) were calculated for NRS and QST parameters as the difference between 6-month follow-up values (T6) and baseline values (T0): Δ=T6−T0.

For descriptive purposes, the magnitude and direction of longitudinal changes were summarized by calculating the median and range of the individual change scores. These change scores were also used to assess the association between longitudinal changes in pain intensity (ΔNRS) and changes in somatosensory function (ΔQST variables).

### 2.5. Statistical Analysis

Statistical analysis was performed using IBM SPSS Statistics 31.0.1.0 (IBM Corp., Armonk, NY, USA).

The distribution of continuous variables was assessed using the Shapiro–Wilk test and visual inspection of quantile–quantile plots and boxplots. As most variables showed significant deviations from normality, including asymmetry and the presence of outliers, non-parametric statistical methods were used.

Categorical variables were described as frequencies and percentages, whereas continuous variables were described using medians and ranges (minimum–maximum), given the distributional characteristics of the data.

Within-subject comparisons between T0 and T6 were performed using the Wilcoxon signed-rank test. Associations between NRS and QST parameters at each time point, as well as correlations between ΔNRS and ΔQST variables, were assessed using Spearman’s rank correlation coefficient (*r_s_*). Because chemotherapy may affect somatosensory function [24,25], exploratory correlation analyses were additionally performed after stratification according to chemotherapy exposure. Owing to the limited number of patients who did not receive chemotherapy (*n* = 14), only the chemotherapy-treated subgroup (*n* = 42) was included in these exploratory analyses.

Given the modest magnitude of the correlations and the potential clinical relevance of distinguishing patients with and without clinically relevant pain (NRS < 4 vs. NRS ≥ 4), additional subgroup analyses were conducted. Group differences in QST parameters were assessed using the Mann–Whitney *U* test. Differences in QST parameters across pain trajectory groups were explored using the Kruskal–Wallis test. When a significant overall group effect was detected, post-hoc pairwise comparisons with Bonferroni correction were performed.

All tests were two-tailed, and statistical significance was set at *p* < 0.05.

Effect sizes for Wilcoxon signed-rank and Mann–Whitney *U* tests were calculated as *r* $=Z/\sqrt{N}$, where *Z* is the test statistic and *N* is the sample size. Values were interpreted according to Cohen’s guidelines [28], with values of 0.10, 0.30, and 0.50 indicating small, medium, and large effects, respectively. Effect sizes for Kruskal–Wallis tests were calculated using ordinal eta-squared (η^2^*_H_*), according to the formula η^2^*_H_* = (*H* − *k* + 1)/(*N* − *k*), where *H* is the Kruskal–Wallis statistic, *k* is the number of groups, and *N* is the sample size. Values of 0.01, 0.06, and 0.14 were interpreted as small, medium, and large effects, respectively [29].

## 3. Results

### 3.1. Study Population Characteristics

The demographic and clinical characteristics of the study population are presented in Table 1. The study included 56 patients with a median age of 52 years. The sample was predominantly female (91.1%), reflecting the high proportion of breast cancer diagnoses (80.4%). Other cancer types included colorectal (10.7%), lung (5.4%), and pancreatic cancer (3.6%). Most patients were diagnosed with stage II (50.0%) or stage III cancer (37.5%), whereas stage I and stage IV accounted for 8.9% and 3.6% of the sample, respectively.

Oncological treatment was heterogeneous, reflecting the multimodal management of cancer patients. The most frequent therapeutic regimen was the combination of chemotherapy and surgery, reported in 23.2% of patients. Across all treatment regimens, chemotherapy was administered to 75.0% of patients, surgery to 87.5%, and radiotherapy to 55.4%. Hormone therapy was administered in 51.8% of cases, while targeted therapy and immunotherapy were less frequent (26.8% and 10.7%, respectively).

Pain trajectory group classification showed that 48.2% of participants were classified as stable low pain, 23.2% as pain increase, and 14.3% each as pain decrease and persistent pain.

### 3.2. Longitudinal Changes in Pain Intensity and Somatosensory Function

Median NRS remained stable over the six-month follow-up period, with a value of 0 at both T0 (range: 0–8) and T6 (range: 0–10). Among thermal sensory thresholds, HPT decreased from 45.43 °C (39.08–52.20) at T0 to 44.44 °C (36.04–54.18) at T6, whereas CDT increased from 24.21 °C (6.90–31.09) to 27.04 °C (7.07–31.06), and CPT increased from 6.81 °C (0.00–23.54) to 11.35 °C (0.00–27.31). Median TSP effect remained unchanged at 2.00, with ranges of 0.00–9.00 at T0 and −2.00–6.00 at T6, while the median CPM effect changed from −1.00 (range: −9.00 to 1.00) at T0 to 0.00 (range: −6.00 to 8.00) at T6.

Significant longitudinal changes were identified in thermal sensory thresholds over the six-month follow-up period (Table 2). HPT significantly decreased over time (30 negative ranks vs. 14 positive ranks; *Z* = −2.241, *p* = 0.025, *r* = 0.34), with a median change score of −0.94 °C (range: −10.32 to 7.86). In contrast, both CDT (12 negative ranks vs. 32 positive ranks; *Z* = −3.291, *p* < 0.001, *r* = 0.50) and CPT (11 negative ranks vs. 27 positive ranks; *Z* = −2.835, *p* = 0.005, *r* = 0.46) significantly increased from T0 to T6, with median change scores of 2.50 °C (range: −11.33 to 15.60) and 4.01 °C (range: −17.38 to 23.91), respectively. The observed decrease in HPT indicates heat pain perception at lower temperatures, whereas the increases in CDT and CPT indicate the perception of cold sensation and cold pain at temperatures closer to the baseline skin temperature (32 °C). The corresponding effect sizes were moderate for HPT and CPT, and large for CDT.

No significant overall changes in pain intensity were observed between T0 and T6. Similarly, no significant longitudinal changes were found in measures related to central pain processing, with negligible effect sizes for both TSP and CPM.

### 3.3. Associations Between Pain Intensity and Somatosensory Function

To investigate whether pain intensity was associated with somatosensory function, correlation analyses were performed between NRS scores and QST parameters at baseline and follow-up, and for longitudinal change scores (Table 3).

At baseline, pain intensity was negatively correlated with cold detection threshold (*r_s_* = −0.371, *p* = 0.009), with higher pain levels being associated with lower CDT values. At 6-month follow-up, pain intensity showed a statistically significant positive correlation with cold pain threshold (*r_s_* = 0.401, *p* = 0.006), with higher pain levels being associated with higher CPT values. No significant correlations were observed between pain intensity and the remaining QST parameters at either time point. Analysis of longitudinal changes further suggested a statistically significant positive association between ΔNRS and ΔCPT (*r_s_* = 0.337, *p* = 0.029). No other statistically significant relationships were identified between ΔNRS and the remaining ΔQST variables.

To investigate whether the observed associations between NRS and thermal sensory thresholds remained after stratification according to chemotherapy exposure, exploratory correlation analyses were performed for chemotherapy-treated patients. The previously observed associations remained statistically significant, including those between NRS and CDT at T0 (*r_s_* = −0.368, *p* = 0.025), NRS and CPT at T6 (*r_s_* = 0.387, *p* = 0.026), and between ΔNRS and ΔCPT (*r_s_* = 0.382, *p* = 0.037). Given the small sample size (*n* = 14), correlation analyses in patients who did not receive chemotherapy were not considered sufficiently reliable and were therefore not included.

### 3.4. Differences According to Clinically Relevant Pain Status

Median HPT, CDT, and CPT values according to clinically relevant pain status at T0 and T6 are presented in Table 4. At baseline, participants with clinically relevant pain showed lower CDT values than those without clinically relevant pain (18.64 °C (10.93–28.74) vs. 25.57 °C (6.90–31.09)), whereas HPT and CPT values were comparable between groups. At follow-up, HPT and CDT values remained similar between groups, whereas participants with clinically relevant pain exhibited higher CPT values (16.03 °C [6.44–27.31] vs. 8.47 °C [0.00–26.96]).

QST outcomes according to clinically relevant pain status at T0 and T6 are presented in Table 5. At T0, participants with clinically relevant pain showed significantly different CDT values compared with those without clinically relevant pain (*U* = 131.0, *Z* = −2.690, *p* = 0.007), with a moderate effect size (*r* = 0.38). At T6, participants with clinically relevant pain exhibited significantly different CPT values compared with those without clinically relevant pain (*U* = 142.0, *Z* = −2.556, *p* = 0.011), also with a moderate effect size (*r* = 0.38). No significant differences were observed for HPT at either time point, or for CDT at T6 and CPT at T0.

### 3.5. Pain Trajectory Groups and Somatosensory Function

To further characterize the relationship between pain and somatosensory function, participants were classified according to pain trajectory groups over the 6-month follow-up period (Table 1), and QST parameters were compared across the four trajectory groups (Table 6). Median NRS scores at T0 and T6 were 0 (0–3) and 0 (0–3) for the stable low pain group, 0 (0–3) and 5 (4–10) for the pain increase group, 5 (4–8) and 0 (0–2) for the pain decrease group, and 5 (4–5) and 6 (5–7) for the persistent pain group, respectively.

A significant difference between pain trajectory groups was observed for baseline CDT (*H*_(3)_= 9.111, *p* = 0.028, η^2^*_H_* = 0.14), indicating a large effect size. Post-hoc pairwise comparisons identified significant differences between the persistent pain and stable low pain groups (*p* = 0.038), the pain decrease and pain increase groups (*p* = 0.044), and the persistent pain and pain increase groups (*p* = 0.006). After Bonferroni adjustment for multiple comparisons, only the difference between the pain increase and persistent pain groups remained statistically significant (adjusted *p* = 0.035). The pain increase group presented higher baseline CDT values than the persistent pain group (mean rank: 33.30 vs. 14.63).

For the remaining thermal measures, no significant between-group differences were identified. HPT and CPT did not differ significantly across pain trajectory groups at either time point, although CPT showed a non-significant trend at T6 (*H*_(3)_ = 6.556, *p* = 0.087, η^2^*_H_* = 0.08).

**Figure 1:**
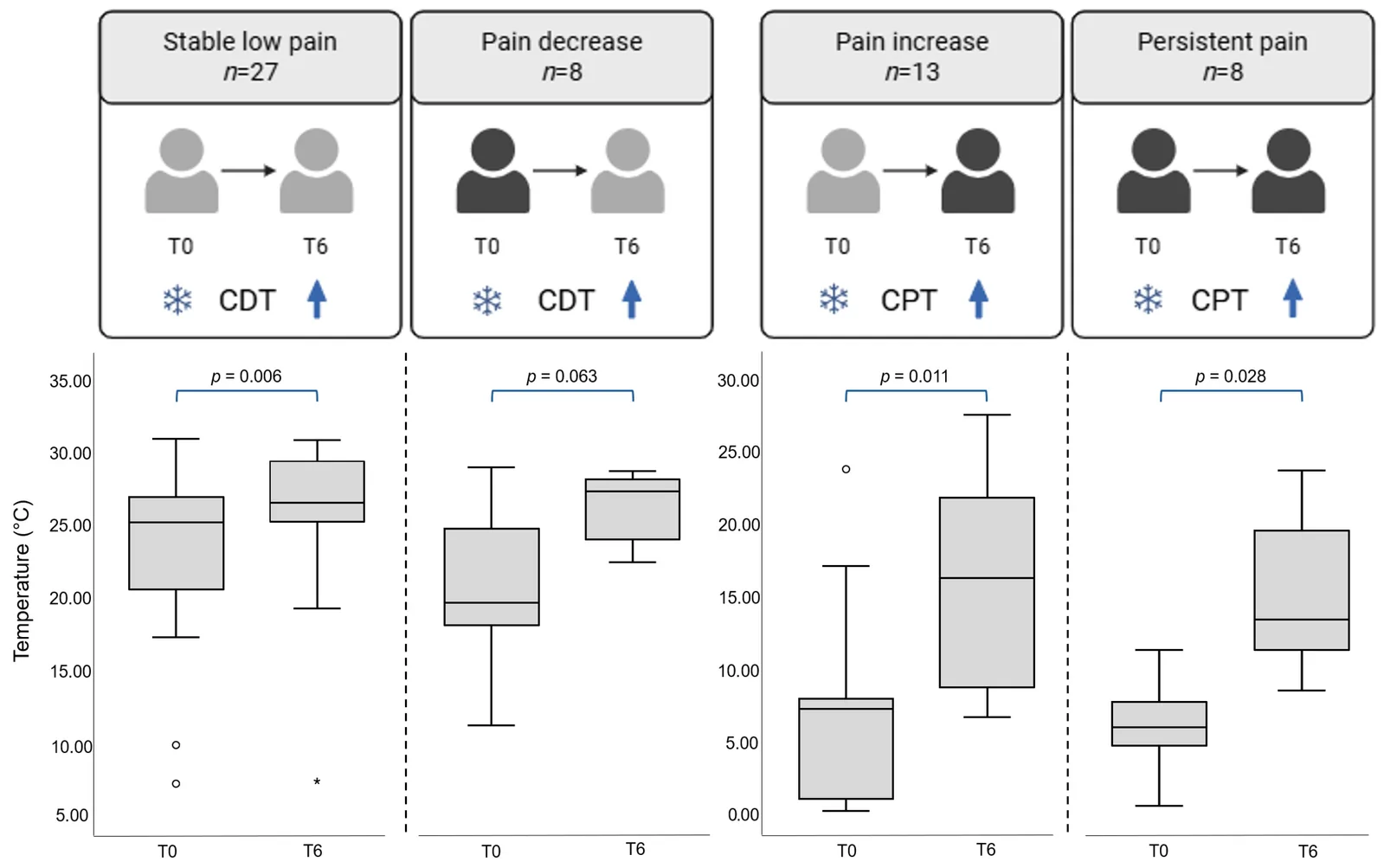
Pain trajectory groups and longitudinal changes in thermal QST measures from T0 to T6. Light grey and dark grey icons represent non-clinically relevant pain (NRS < 4) and clinically relevant pain (NRS ≥ 4), respectively. Arrows indicate the direction of significant longitudinal changes within each trajectory group. The snowflake represents cold thermal measures. Boxes represent the median and interquartile range (IQR), whiskers represent 1.5 × IQR, and circles/asterisks indicate outliers. CDT, Cold Detection Threshold; CPT, Cold Pain Threshold; T0, baseline assessment; T6, six-month follow-up assessment. Created in https://BioRender.com.

Following the between-group comparisons presented in Table 6, exploratory within-group analyses were conducted using the Wilcoxon signed-rank test to assess longitudinal changes in NRS and QST parameters within each pain trajectory group. The significant findings are summarized in Figure 1. An increase in CDT was observed in the stable low pain group (*p* = 0.006), whereas increases in CPT were observed in the pain increase (*p* = 0.011) and persistent pain groups (*p* = 0.028). Changes in NRS were identified in all trajectory groups except the stable low pain group (*p* ≤ 0.023). No significant longitudinal changes were observed in HPT, TSP, or CPM. Detailed statistical results are presented in the Supplementary Material (Supplementary Tables S1–S4).

Exploratory correlation analyses were conducted within each pain trajectory group to investigate associations between NRS and QST parameters at baseline and follow-up, and for longitudinal change scores. A limited number of statistically significant correlations were identified, varying across groups and time points, with no consistent pattern emerging across trajectories. Given the small sample size within each subgroup and the exploratory nature of these analyses, detailed results are presented in the Supplementary Material (Supplementary Tables S5–S8).

## 4. Discussion

The main finding of the present study was the identification of significant longitudinal changes in thermal sensory thresholds during the first six months following cancer diagnosis, particularly involving cold sensory processing, whereas pain intensity remained stable and no significant longitudinal changes were observed in TSP or CPM. These findings contribute to the limited longitudinal evidence regarding somatosensory function from cancer diagnosis and prior to treatment exposure [23].

Specifically, HPT decreased over time, while CDT and CPT increased, indicating greater sensitivity to both noxious heat and cold stimuli. The decrease in HPT suggests that painful heat stimuli were perceived at lower temperatures over time, consistent with increased heat pain sensitivity. In contrast, higher CDT values indicate that cold sensations were detected with smaller decreases in temperature, whereas higher CPT values indicate that cold pain was perceived at less extreme cold temperatures. Collectively, these findings suggest alterations in thermal sensory processing during the first months following cancer diagnosis. Previous studies using QST in neuropathic and chronic pain conditions have similarly demonstrated that thermal modalities are particularly sensitive to alterations in small-fiber function and peripheral nociceptive pathways [30,31,32,33]. Since thermal detection and pain perception are primarily mediated by Aδ and C fibers [13], these findings may indicate subtle alterations in small-fiber sensory function occurring during the first months after diagnosis and treatment initiation.

Several biological mechanisms may contribute to these findings. Cancer itself may induce peripheral sensitization through inflammation, release of algogenic mediators, and neuro-immune interactions [16,17], while, in advanced cases, disruption of bone homeostasis may further contribute to pain sensitization [34]. In addition, some oncological treatments, particularly neurotoxic chemotherapy agents, have been associated with sensory dysfunction and chemotherapy-induced peripheral neuropathy [24,25]. Although the present study was not designed to investigate treatment-specific effects, the combination of different treatment modalities precluded attributing these sensory changes to a single mechanism and highlights the need for future studies exploring sensory trajectories according to treatment regimen and cumulative exposure.

In contrast, no significant longitudinal alterations were identified in TSP and CPM, suggesting that in the present study no measurable changes in psychophysical proxy measures of facilitatory and inhibitory pain modulation were detected during the first six months of follow-up. Previous studies have shown inconsistent findings regarding central pain modulatory mechanisms in oncological populations, suggesting that their role may depend on disease stage, treatment, pain phenotype or psychosocial factors [22,35,36]. Current evidence also supports that central sensitization often develops after prolonged exposure to persistent nociceptive input rather than during the early stages of disease [37,38]. For example, Arendt-Nielsen et al. reported that manifestations of central sensitization tend to become more evident in chronic pain conditions with longer exposure to persistent nociceptive input. Similarly, Curatolo highlighted that measurable alterations in central pain processing are more frequently observed in established chronic pain states. Taken together, the present findings indicate that peripheral sensory alterations were detectable in the absence of measurable changes in psychophysical proxy measures of central pain modulation over the six-month follow-up. One possible interpretation is that peripheral sensory alterations may precede detectable changes in central pain modulation during the early stages of cancer-related pain. However, as only two assessment time points were available, alternative temporal patterns cannot be excluded, including transient changes in central pain modulation between assessments or the emergence of such changes later in the disease or treatment trajectory.

Although longitudinal changes were observed across several thermal QST measures, the most consistent associations with pain involved cold-related modalities. Higher pain levels at T0 were associated with lower CDT values, whereas at T6, higher pain intensity was associated with higher CPT values. Furthermore, longitudinal changes in pain intensity were positively correlated with changes in CPT. Although the correlations were modest, their consistency across analyses suggests that cold sensory processing may be particularly relevant to the experience and evolution of cancer-related pain. This observation is consistent with Malmström et al., who, in a small clinical study, reported that cold thermal thresholds were among the most informative QST measures for characterizing persistent pain [39].

The negative correlation between CDT and NRS at T0 may indicate that patients reporting higher pain levels already presented subtle alterations in innocuous cold perception. Such findings are consistent with studies demonstrating that abnormalities in thermal detection thresholds can reflect early dysfunction of small sensory fibers before overt neuropathic symptoms become clinically apparent [12,32,33]. Baron et al. and Maier et al. reported that thermal sensory abnormalities frequently characterize distinct neuropathic sensory phenotypes and may precede more pronounced pain manifestations. In the context of cancer, these alterations may reflect early peripheral sensitization or neuro-immune mechanisms related to the tumor microenvironment [16,40].

Interestingly, the relationship observed at T6 involved CPT rather than CDT. Because CPT reflects the processing of noxious cold stimuli, this finding may suggest that, over time, pain becomes increasingly associated with nociceptive rather than purely sensory thermal pathways [26]. The positive correlation between ΔNRS and ΔCPT is consistent with this interpretation, indicating that patients who experienced worsening pain also tended to exhibit greater increases in cold pain sensitivity. Together, these findings suggest different components of cold sensory processing may be associated with pain intensity at different stages during the first months following cancer diagnosis. As CDT primarily reflects Aδ fiber-mediated innocuous cold detection, whereas CPT reflects activation of Aδ and C nociceptive afferents during cold pain perception, one possible interpretation is that the relationship between pain intensity and cold sensory processing may involve different sensory mechanisms over time [30].

Beyond the potential differential involvement of peripheral sensory afferents, alterations in endogenous pain modulation may also contribute to the observed association between pain intensity and cold pain sensitivity. Within the framework of the Gate Control Theory and its subsequent developments in endogenous pain modulation, ongoing nociceptive input may be expected to activate descending inhibitory pathways and attenuate the perception of subsequent noxious stimuli [41,42]. However, the present study showed that higher pain intensity was associated with increased cold pain sensitivity, as reflected by higher CPT values. One possible explanation is that descending inhibitory mechanisms may already be impaired or insufficiently recruited in some patients, limiting their ability to modulate nociceptive input despite the absence of detectable alterations in CPM [43,44]. Alternatively, these findings may reflect inter-individual differences in pain sensitivity, whereby some individuals exhibit an inherently greater responsiveness to nociceptive stimuli [45]. Finally, peripheral sensitization driven by tumor-related neuro-immune interactions may increase peripheral afferent input to an extent that exceeds endogenous inhibitory capacity, contributing to enhanced cold pain sensitivity [46,47].

These associations remained significant after restricting the analyses to chemotherapy-treated patients, suggesting that the observed relationship between NRS and thermal sensory function may also be present in this group. However, because correlation analyses were not performed in patients who did not receive chemotherapy due to the limited sample size, it was not possible to determine whether these associations are specific to chemotherapy-treated patients. Previous evidence has shown that chemotherapy-induced peripheral neuropathy is associated with alterations in thermal detection and pain thresholds [24,25], providing a plausible biological basis for the associations observed in the present study. Future studies including larger numbers of patients not exposed to chemotherapy are needed to determine whether chemotherapy influences these associations.

The trajectory-based analyses further reinforced the relevance of cold sensory processing. Among all QST variables evaluated, CDT at T0 was the only parameter that significantly differed between the pain trajectory groups. Post-hoc pairwise comparisons with Bonferroni correction indicated that patients belonging to the pain increase trajectory had significantly higher baseline CDT values than those with persistent pain. This finding suggests that cold detection function assessed before pain trajectory groups become established may capture clinically meaningful differences between sensory phenotypes. Previous studies have proposed that QST-derived sensory profiles may help identify subgroups of patients characterized by different underlying pain mechanisms and clinical outcomes [9,20,48]. Vollert et al. demonstrated that sensory phenotyping based on QST can distinguish clinically relevant neuropathic pain subgroups, while Vaegter and Graven-Nielsen showed that pain modulatory phenotypes are associated with differences in pain sensitivity. The present findings extend this concept to an oncological population and suggest that differences in cold sensory detection assessed at baseline may be associated with distinct patterns of pain evolution. Interestingly, patients in the pain increase trajectory exhibited higher baseline CDT values than those in the persistent pain trajectory, despite not yet reporting clinically relevant pain. As higher CDT values indicate that cold sensation is detected with smaller decreases in temperature, this finding suggests that subtle differences in cold sensory processing may already be present before clinically relevant pain develops. This observation raises the possibility that subtle alterations in cold sensory processing may precede the clinical worsening of pain. Although evidence supporting the prognostic value of baseline QST in oncology remains limited, similar approaches have shown promise in predicting pain outcomes across several non-cancer chronic pain conditions, particularly musculoskeletal disorders [49]. If confirmed in larger longitudinal studies of cancer populations, baseline QST may contribute to the early identification of patients at increased risk of pain progression [23].

Additional within-trajectory analyses reinforced this interpretation. Patients in the stable low pain trajectory group demonstrated a significant increase in CDT over time, whereas increases in CPT were observed only in the pain increase and persistent pain trajectory groups. These findings suggest that distinct sensory processes may underlie different patterns of pain evolution. While changes in cold detection may occur even in patients who remain largely pain-free, increasing sensitivity to cold pain appears to be more characteristic of trajectories associated with clinically relevant pain. Although exploratory, these observations reinforce the potential value of cold-related QST measures for characterizing different patterns of pain evolution following cancer diagnosis.

This study has some limitations. First, the relatively small sample size, particularly within the pain trajectory subgroups, limited the statistical power of subgroup analyses, therefore, these findings should be considered exploratory and require confirmation in larger cohorts. Second, the predominance of breast cancer patients may limit the generalizability of the findings to other oncological populations. Third, heterogeneity in cancer type, disease stage, and treatment exposure may have increased between-participant variability in somatosensory outcomes and either diluted or obscured effects specific to particular clinical subgroups. Moreover, because participants received different and frequently combined treatment modalities, it was not possible to determine whether the observed sensory changes were primarily related to the underlying disease, disease stage, specific treatment-related effects (e.g., chemotherapy-induced neuropathic pain or hormonal influences associated with hormone therapy), or their combined effects. Furthermore, QST was performed at a remote body site (the volar forearm), which may better reflect generalized somatosensory function than local sensory alterations at the primary pain region. Therefore, the observed thermal sensory changes should not be directly interpreted as evidence of peripheral sensitization at the site of cancer-related pain. In addition, only two assessment time points were available, precluding characterization of the temporal evolution of pain modulatory mechanisms and the identification of potential transient changes between assessments. Finally, the absence of longitudinal alterations in TSP and CPM should be interpreted with caution, as these psychophysical proxy measures are characterized by considerable inter-individual variability and only moderate reliability. Although all assessments were conducted under standardized conditions in a private, quiet room to minimize environmental distractions and maximize participant comfort, psychophysical measures remain inherently susceptible to cognitive and psychological influences. Moreover, although no global adjustment for multiple comparisons was applied, the statistical analyses were performed on a predefined set of clinically relevant QST outcomes selected based on their biological plausibility and established role in assessing peripheral and central pain processing. Nevertheless, the number of statistical tests performed may have increased the risk of type I error, therefore, statistically significant findings should be interpreted with appropriate caution and confirmed in larger independent cohorts. Nevertheless, the longitudinal design and comprehensive assessment of somatosensory function from diagnosis to follow-up provide valuable insights into the early evolution of cancer-related pain.

Overall, these preliminary results suggest that alterations in thermal sensory processing may contribute to the characterization of sensory profiles associated with different patterns of cancer-related pain. Although no evidence of longitudinal changes in central pain modulation was observed, cold-related QST measures consistently showed the strongest associations with pain intensity, pain trajectory groups, and longitudinal pain evolution, supporting their potential clinical relevance. Further longitudinal studies including larger and more diverse cohorts are warranted to confirm these findings and clarify the role of QST in the assessment of cancer-related pain.

## 5. Conclusions

This study demonstrated that thermal sensory function undergoes measurable longitudinal changes during the first six months following cancer diagnosis. Among the QST measures evaluated, cold-related modalities consistently showed the strongest associations with pain intensity and pain trajectory groups. These results suggest that alterations in cold sensory processing may represent an early feature of cancer-related pain. Overall, these findings suggest that thermal QST, particularly cold-related measures, may be useful for characterizing sensory profiles in cancer patients, and support further investigation into their potential clinical relevance.

## Figures and Tables

**Table 1:** Baseline demographic and clinical characteristics of the study population.

Study Population Characteristics	*n* (%)
Gender	
Men	5 (8.9)
Women	51 (91.1)
Age, median (min–max)	52 (27–84)
Men	61 (49–84)
Women	51 (27–79)
Cancer type	
Breast	45 (80.4)
Colorectal	6 (10.7)
Lung	3 (5.4)
Pancreas	2 (3.6)
Cancer stage at diagnosis	
I	5 (8.9)
II	28 (50.0)
III	21 (37.5)
IV	2 (3.6)
Therapeutic regimens	
Chemotherapy + Surgery	13 (23.2)
Chemotherapy	6 (10.7)
Surgery	5 (8.9)
Hormone therapy + Radiotherapy + Surgery	5 (8.9)
Chemotherapy + Immunotherapy + Surgery	4 (7.1)
Chemotherapy + Hormone therapy + Radiotherapy + Surgery	4 (7.1)
Chemotherapy + Radiotherapy + Surgery	3 (5.4)
Chemotherapy + Targeted therapy + Surgery	3 (5.4)
Chemotherapy + Immunotherapy	2 (3.6)
Chemotherapy + Targeted therapy	2 (3.6)
Chemotherapy + Hormone therapy + Surgery	2 (3.6)
Chemotherapy + Hormone therapy + Targeted therapy + Surgery	2 (3.6)
Hormone therapy + Surgery	1 (1.8)
Radiotherapy + Surgery	1 (1.8)
Chemotherapy + Hormone therapy + Targeted Therapy + Radiotherapy + Surgery	1 (1.8)
Targeted Therapy	1 (1.8)
Radiotherapy	1 (1.8)
Pain trajectory group	
Stable low pain	27 (48.2)
Pain increase	13 (23.2)
Pain decrease	8 (14.3)
Persistent pain	8 (14.3)

**Table 2:** Longitudinal changes in NRS and QST parameters between T0 and T6.

Variable	Negative Ranks (*n*)	Positive Ranks (*n*)	Ties (*n*)	*Z*	*p*-Value	*r*
NRS	11	21	24	−1.494	0.135	0.26
HPT	30	14	0	−2.241	0.025 *	0.34
CDT	12	32	0	−3.291	<0.001 *	0.50
CPT	11	27	4	−2.835	0.005 *	0.46
TSP effect	19	17	9	−0.285	0.776	0.05
CPM effect	15	23	7	−0.337	0.736	0.05

Comparisons between T0 and T6 were performed using the Wilcoxon signed-rank test. Negative ranks indicate lower values at T6 compared with T0, whereas positive ranks indicate higher values at T6 compared with T0. Ties indicate no change between T0 and T6. CDT, Cold Detection Threshold; CPM, Conditioned Pain Modulation; CPT, Cold Pain Threshold; HPT, Heat Pain Threshold; NRS, Numeric Rating Scale; T0, baseline assessment; T6, six-month follow-up assessment; TSP, Temporal Summation of Pain. *Z*, standardized test statistic; *r*, effect size (*r* = *Z*/√*N*). * *p* < 0.05.

**Table 3:** Correlations between NRS and QST parameters at baseline (NRS T0 vs. QST T0), at 6-month follow-up (NRS T6 vs. QST T6), and between longitudinal changes in pain intensity and QST parameters (ΔNRS vs. ΔQST).

11 Pain Intensity	11 QST Measures	*r_s_*	*p*-Value
	HPT T0	0.175	0.230
	CDT T0	−0.371	0.009 *
NRS T0	CPT T0	−0.057	0.697
	TSP effect T0	−0.187	0.198
	CPM effect T0	0.156	0.283
	HPT T6	−0.265	0.069
	CDT T6	0.090	0.541
NRS T6	CPT T6	0.401	0.006 *
	TSP effect T6	0.012	0.936
	CPM effect T6	−0.070	0.633
	Δ HPT	0.067	0.667
	Δ CDT	−0.200	0.192
Δ NRS	Δ CPT	0.337	0.029 *
	Δ TSP effect	−0.043	0.780
	Δ CPM effect	−0.030	0.843

Correlations were assessed using Spearman’s correlation coefficient (*r_s_*). CDT, Cold Detection Threshold; CPM, Conditioned Pain Modulation; CPT, Cold Pain Threshold; HPT, Heat Pain Threshold; NRS, Numeric Rating Scale; QST, Quantitative Sensory Testing; T0, baseline assessment; T6, six-month follow-up assessment; TSP, Temporal Summation of Pain. * *p* < 0.05.

**Table 4:** Thermal QST values according to clinically relevant pain status at T0 and T6.

Variable	T0 NRS < 4	T0 NRS ≥ 4	T6 NRS < 4	T6 NRS ≥ 4
HPT (°C)	45.45 (39.08–52.20)	45.43 (40.81–51.74)	44.66 (36.04–54.18)	43.52 (37.70–48.00)
CDT (°C)	25.57 (6.90–31.09)	18.64 (10.93–28.74)	26.29 (7.07–30.50)	27.36 (8.11–31.06)
CPT (°C)	6.88 (0.00–23.54)	6.67 (0.00–22.25)	8.47 (0.00–26.96)	16.03 (6.44–27.31)

Values are presented as median (minimum–maximum). Participants were classified according to clinically relevant pain status using the NRS: NRS < 4 (non-clinically relevant pain) and NRS ≥ 4 (clinically relevant pain). Group sizes were as follows: T0, NRS < 4 (*n* = 40) and NRS ≥ 4 (*n* = 16); T6, NRS < 4 (*n* = 35) and NRS ≥ 4 (*n* = 21). CDT, Cold Detection Threshold; CPT, Cold Pain Threshold; HPT, Heat Pain Threshold; NRS, Numeric Rating Scale; QST, Quantitative Sensory Testing; T0, baseline assessment; T6, six-month follow-up assessment.

**Table 5:** Comparison of HPT, CDT, and CPT according to clinically relevant pain status (NRS < 4 vs. NRS ≥ 4) at T0 and T6.

Variable	Time	NRS < 4	NRS ≥ 4	*U*	*Z*	*p*-Value	*r*
HPT	T0	24.76	25.53	247.0	−0.174	0.862	0.03
	T6	27.47	19.97	189.5	−1.813	0.070	0.26
CDT	T0	28.65	16.73	131.0	−2.690	0.007 *	0.38
	T6	24.62	24.32	272.0	−0.074	0.941	0.01
CPT	T0	25.07	24.83	252.5	−0.054	0.957	0.01
	T6	19.26	29.53	142.0	−2.556	0.011 *	0.38

Values are presented as mean ranks. Comparisons between participants with and without clinically relevant pain were performed using the Mann–Whitney *U* test. Higher mean ranks indicate relatively higher values of the corresponding variable. Group sizes were as follows: T0, NRS < 4 (*n* = 40) and NRS ≥ 4 (*n* = 16); T6, NRS < 4 (*n* = 35) and NRS ≥ 4 (*n* = 21). CDT, Cold Detection Threshold; CPT, Cold Pain Threshold; HPT, Heat Pain Threshold; NRS, Numeric Rating Scale; T0, baseline assessment; T6, six-month follow-up assessment. *U*, Mann–Whitney *U* statistic; *Z*, standardized test statistic; *r*, effect size (*r* = *Z*/√*N*). * *p* < 0.05.

**Table 6:** Comparison of HPT, CDT, and CPT across pain trajectory groups at T0 and T6.

Variable	Time	Stable Low Pain	Pain Increase	Pain Decrease	Persistent Pain	*H*	*p*-Value	η^2^*_H_*
HPT	T0	24.85	24.55	22.71	28.00	0.544	0.909	0.00
	T6	27.34	18.71	27.86	22.14	3.561	0.313	0.01
CDT	T0	26.71	33.30	19.14	14.63	9.111	0.028 *	0.14
	T6	25.50	28.92	21.86	16.43	3.883	0.274	0.02
CPT	T0	25.67	23.65	30.57	19.81	2.280	0.516	0.00
	T6	19.05	29.67	19.86	29.29	6.556	0.087	0.08

Values are presented as mean ranks. Differences across groups were explored using the Kruskal-Wallis test. * Bonferroni-adjusted post hoc comparisons revealed a significant difference only between the pain increase and persistent pain groups (adjusted *p* = 0.035). CDT, Cold Detection Threshold; CPT, Cold Pain Threshold; HPT, Heat Pain Threshold; T0, baseline assessment; T6, six-month follow-up assessment. *H*, Kruskal–Wallis test statistic; η^2^*_H_*, effect size (η^2^*_H_* = (*H* − *k* + 1)/(*N* − *k*)) * *p* < 0.05.

## Data Availability

The data that support the findings of this study are available from the corresponding author, R.M. and A.L.T., upon reasonable request.

## Supplementary Materials

The following supporting information can be downloaded at: https://www.mdpi.com/article/10.3390/biomedicines14081840/s1, Table S1A: Descriptive statistics for the Stable Low Pain trajectory group, Table S1B: Changes in NRS and QST parameters between T0 and T6 in the Stable Low Pain trajectory group, Table S2A: Descriptive statistics for the Pain Increase trajectory group, Table S2B: Changes in NRS and QST parameters between T0 and T6 in the Pain Increase trajectory group, Table S3A: Descriptive statistics for the Pain Decrease trajectory group, Table S3B: Changes in NRS and QST parameters between T0 and T6 in the Pain Decrease trajectory group, Table S4A: Descriptive statistics for the Persistent Pain trajectory group, Table S4B: Changes in NRS and QST parameters between T0 and T6 in the Persistent Pain trajectory group, Table S5: Correlations between pain intensity and QST parameters at baseline (NRS T0 vs. QST T0), at 6-month follow-up (NRS T6 vs. QST T6), and between longitudinal changes in pain intensity and QST parameters (ΔNRS vs. ΔQST) within the Stable Low Pain Trajectory group, Table S6: Correlations between pain intensity and QST parameters at baseline (NRS T0 vs. QST T0), at 6-month follow-up (NRS T6 vs. QST T6), and between longitudinal changes in pain intensity and QST parameters (ΔNRS vs. ΔQST) within the Pain Increase Trajectory Group, Table S7: Correlations between pain intensity and QST parameters at baseline (NRS T0 vs. QST T0), at 6-month follow-up (NRS T6 vs. QST T6), and between longitudinal changes in pain intensity and QST parameters (ΔNRS vs. ΔQST) within the Pain Decrease trajectory group, Table S8: Correlations between pain intensity and QST parameters at baseline (NRS T0 vs. QST T0), at 6-month follow-up (NRS T6 vs. QST T6), and between longitudinal changes in pain intensity and QST parameters (ΔNRS vs. ΔQST) within the Persistent Pain trajectory group.

## Abbreviations

The following abbreviations are used in this manuscript:

Aδ	A-delta fibers
CDT	Cold Detection Threshold
CPM	Conditioned Pain Modulation
CPT	Cold Pain Threshold
HPT	Heat Pain Threshold
IPO Porto	Portuguese Institute of Oncology of Porto
NRS	Numeric Rating Scale
QST	Quantitative Sensory Testing
SPSS	Statistical Package for the Social Sciences
T0	Baseline assessment
T6	Six-month follow-up assessment
TRP	Transient Receptor Potential
TSP	Temporal Summation of Pain
