# Supplementary material for: Longitudinal Changes in Somatosensory Function and Their Association with Pain Trajectory Groups in a Portuguese Cancer Center Cohort
Source: Biomedicines. 2026 Aug 15;14(8):1840. doi: 10.3390/biomedicines14081840 (PMC13509572; doi:10.3390/biomedicines14081840)
Supplement: Supplementary file 1 [file biomedicines-14-01840-s001.zip › biomedicines-4480556-supplementary.pdf]

Table S1. A. Descriptive statistics for the Stable Low Pain trajectory group.

Variable	Median (min–max) T0	Median (min–max) T6
NRS	0 (0–3)	0 (0–3)
HPT	45.34 (39.08–52.20)	44.63 (36.04–54.18)
CDT	25.12 (6.90–30.69)	26.65 (7.07–30.60)
CPT	6.88 (0.00–22.20)	8.34 (0.00–26.96)
TSP effect	2.5 (0–7)	2.0 (−2–6)
CPM effect	−0.5 (−9–1)	0.0 (−5–3)

Table S1. B. Changes in NRS and QST parameters between T0 and T6 in the Stable Low Pain trajectory group.

Variable	Negative Ranks (*n*)	Positive Ranks (*n*)	Ties (*n*)	*Z*	*p*-value	*r*
NRS	3	2	22	−0.138	0.890	0.06
HPT	15	6	0	−1.582	0.114	0.35
CDT	5	16	0	−2.746	0.006	0.60
CPT	6	10	3	−1.138	0.255	0.26
TSP effect	11	6	5	−1.272	0.203	0.31
CPM effect	7	9	6	−0.260	0.795	0.07

Comparisons between T0 and T6 were performed using the Wilcoxon signed-rank test. Negative ranks indicate lower values at T6 compared with T0, whereas positive ranks indicate higher values at T6 compared with T0. Ties indicate no change between T0 and T6. Effect size was calculated as r = Z/√N.

Table S2. A. Descriptive statistics for the Pain Increase trajectory group.

Variable	Median (min–max) T0	Median (min–max) T6
NRS	0 (0–3)	5 (4–10)
HPT	45.45 (40.50–49.89)	43.56 (37.70–46.21)
CDT	27.53 (18.48–31.09)	28.35 (8.11–31.06)
CPT	6.91 (0.00–23.54)	17.51 (6.44–27.31)
TSP effect	1.5 (0–9)	2.0 (0–5)
CPM effect	−1.0 (−6–1)	−0.5 (−6–1)

Table S2. B. Changes in NRS and QST parameters between T0 and T6 in the Pain Increase trajectory group.

Variable	Negative Ranks (*n*)	Positive Ranks (*n*)	Ties (*n*)	*Z*	*p*-value	*r*
NRS	0	13	0	−3.204	0.001	0.89
HPT	6	3	0	−1.007	0.314	0.34
CDT	3	6	0	−0.533	0.594	0.18
CPT	1	8	0	−2.547	0.011	0.85

Variable	Negative Ranks (*n*)	Positive Ranks (*n*)	Ties (*n*)	*Z*	*p*-value	*r*
TSP effect	3	5	1	0.000	1.000	0.00
CPM effect	3	5	1	−0.281	0.779	0.10

Comparisons between T0 and T6 were performed using the Wilcoxon signed-rank test. Negative ranks indicate lower values at T6 compared with T0, whereas positive ranks indicate higher values at T6 compared with T0. Ties indicate no change between T0 and T6. Effect size was calculated as r = Z/√N.

Table S3. A. Descriptive statistics for the Pain Decrease trajectory group.

Variable	Median (min–max) T0	Median (min–max) T6
NRS	5 (4–8)	0 (0–2)
HPT	45.23 (42.99–50.95)	44.75 (41.67–50.85)
CDT	19.37 (10.93–28.74)	27.09 (22.17–28.47)
CPT	9.13 (0.00–22.25)	9.02 (0.00–22.70)
TSP effect	2.0 (0–4)	3.0 (1–6)
CPM effect	0.0 (−2–0)	0.0 (−3–8)

Table S3. B. Changes in NRS and QST parameters between T0 and T6 in the Pain Decrease trajectory group.

Variable	Negative Ranks (*n*)	Positive Ranks (*n*)	Ties (*n*)	*Z*	*p*-value	*r*
NRS	8	0	0	−2.585	0.010	0.91
HPT	4	3	0	−0.676	0.499	0.26
CDT	2	5	0	−1.859	0.063	0.70
CPT	4	3	0	−0.338	0.735	0.13
TSP effect	1	5	1	−1.378	0.168	0.52
CPM effect	2	5	0	−0.512	0.609	0.19

Negative ranks indicate lower values at T6 compared with T0, whereas positive ranks indicate higher values at T6 compared with T0. Comparisons between T0 and T6 were performed using the Wilcoxon signed-rank test. Effect size was calculated as r = Z/√N.

Table S4. A. Descriptive statistics for the Persistent Pain trajectory group.

Variable	Median (min–max) T0	Median (min–max) T6
NRS	5 (4–5)	6 (5–7)
HPT	46.18 (40.81–51.74)	43.52 (38.32–48.00)
CDT	17.73 (12.10–26.98)	24.51 (14.00–28.40)
CPT	5.65 (0.00–11.06)	13.16 (8.27–23.45)
TSP effect	1.5 (0–7)	1.0 (0–4)
CPM effect	−1.0 (−1–1)	−1.0 (−2–0)

Table S4. B. Changes in NRS and QST parameters between T0 and T6 in the Persistent Pain trajectory group.

Variable	Negative Ranks (*n*)	Positive Ranks (*n*)	Ties (*n*)	*Z*	*p*-value	*r*
NRS	0	6	2	−2.271	0.023	0.93
HPT	5	2	0	−0.845	0.398	0.32
CDT	2	5	0	−1.014	0.310	0.38
CPT	0	6	1	−2.201	0.028	0.90
TSP effect	4	1	2	−0.948	0.343	0.42
CPM effect	3	4	0	−3.78	0.705	0.14

Comparisons between T0 and T6 were performed using the Wilcoxon signed-rank test. Negative ranks indicate lower values at T6 compared with T0, whereas positive ranks indicate higher values at T6 compared with T0. Ties indicate no change between T0 and T6. Effect size was calculated as r = Z/√N.

Table S5. Correlations between pain intensity and QST parameters at baseline (NRS T0 vs QST T0), at 6-month follow-up (NRS T6 vs QST T6), and between longitudinal changes in pain intensity and QST parameters (ΔNRS vs ΔQST) within the Stable Low Pain Trajectory group.

Stable Low Pain Trajectory Group		*rs*	*p*-value
NRS T0	HPT T0	0.397	0.055
	CDT T0	−0.328	0118
	CPT T0	−0.274	0.194
	TSP effect T0	−0.402	0.051
	CPM effect T0	0.414	0.044
NRS T6	HPT T6	0.174	0.437
	CDT T6	0.274	0.217
	CPT T6	−0.140	0.557
	TSP effect T6	0.354	0.097
	CPM effect T6	−0.421	0.045
Δ NRS	Δ HPT	0.188	0.415
	Δ CDT	−0.031	0.893
	Δ CPT	−0.123	0.615
	Δ TSP effect	0.076	0.738
	Δ CPM effect	−0.147	0.514

Correlations were assessed using Spearman's correlation coefficient (rs).

Table S6. Correlations between pain intensity and QST parameters at baseline (NRS T0 vs QST T0), at 6-month follow-up (NRS T6 vs QST T6), and between longitudinal changes in pain intensity and QST parameters (ΔNRS vs ΔQST) within the Pain Increase Trajectory Group.

Pain Increase Trajectory Group		*rs*	*p*-value
NRS T0	HPT T0	0.311	0.381
	CDT T0	0.588	0.074
	CPT T0	0.018	0.962

Pain Increase Trajectory Group		*rs*	***p*-value**
	TSP effect T0	−0.045	0.902
	CPM effect T0	−0.198	0.584
NRS T6	HPT T6	−0.247	0.439
	CDT T6	0.272	0.392
	CPT T6	0.193	0.549
	TSP effect T6	−0.028	0.932
	CPM effect T6	0.468	0.125
Δ NRS	Δ HPT	0.596	0.090
	Δ CDT	0.220	0.569
	Δ CPT	0.018	0.963
	Δ TSP effect	0.273	0.477
	Δ CPM effect	0.470	0.202

Correlations were assessed using Spearman's correlation coefficient (rs).

Table S7. Correlations between pain intensity and QST parameters at baseline (NRS T0 vs QST T0), at 6-month follow-up (NRS T6 vs QST T6), and between longitudinal changes in pain intensity and QST parameters (ΔNRS vs ΔQST) within the Pain Decrease trajectory group.

Pain Decrease Trajectory Group		*rs*	***p*-value**
NRS T0	HPT T0	0.319	0.486
	CDT T0	0.000	1.000
	CPT T0	0.120	0.799
	TSP effect T0	0.406	0.366
	CPM effect T0	−0.176	0.705
NRS T6	HPT T6	−0.408	0.363
	CDT T6	0.000	1.000
	CPT T6	0.408	0.363
	TSP effect T6	0.312	0.496
	CPM effect T6	0.624	0.135
Δ NRS	Δ HPT	0.158	0.736
	Δ CDT	0.236	0.610
	Δ CPT	0.433	0.331
	Δ TSP effect	0.557	0.194
	Δ CPM effect	0.175	0.707

Correlations were assessed using Spearman's correlation coefficient (rs).

Table S8. Correlations between pain intensity and QST parameters at baseline (NRS T0 vs QST T0), at 6-month follow-up (NRS T6 vs QST T6), and between longitudinal changes in pain intensity and QST parameters (ΔNRS vs ΔQST) within the Persistent Pain trajectory group.

Persistent Pain Trajectory Group		*rs*	***p*-value**

Persistent Pain Trajectory Group		*rs*	*p*-value
NRS T0	HPT T0	0.756	0.300
	CDT T0	−0.126	0.766
	CPT T0	−0.252	0.547
	TSP effect T0	−0.715	0.046
	CPM effect T0	0.436	0.280
NRS T6	HPT T6	−0.265	0.566
	CDT T6	0.945	0.001
	CPT T6	0.605	0.105
	TSP effect T6	−0.154	0.742
	CPM effect T6	−0.024	0.960
Δ NRS	Δ HPT	−0.60	0.899
	Δ CDT	0.239	0.606
	Δ CPT	0.478	0.278
	Δ TSP effect	0.091	0.846
	Δ CPM effect	0.000	1.000

Correlations were assessed using Spearman's correlation coefficient (rs).
